# A multi-centric, single-blinded, randomized, parallel-group study to evaluate the effectiveness of nasoalveolar moulding treatment in non-syndromic patients with complete unilateral cleft lip, alveolus and palate (NAMUC study): a study protocol for a randomized controlled trial

**DOI:** 10.1186/s13063-024-08229-z

**Published:** 2024-07-04

**Authors:** Badri Thiruvenkatachari, Krishnamurthy Bonanthaya, Anne Marie Kuijpers Jagtman, Jonathan Sandler, Rajesh S Powar, Syed Altaf Hussain, B. Subramaniyan, Nitin Bhola, Hari Kishore Bhat, Varun Ramachandra, Sylvia Jayakumar, Puneet Batra, Subhiksha Chakkaravarthi, V. Thailavathy, Manoj Prathap, Thulasiram Elumalai, Karthika Nambiar, Charanya Vijayakumar, Ravi Kumar Mahajan, Sundereshwer Chander Sood, Sukhdeep Singh Kahlon, Shruti Bijapur, Ranjit Haridas Kamble, K. M. Keluskar, Amit Nilgar, Faizan Ahmed Khan, Devika Das, Swetha Sridhar, Adrija Buch, Shuba Kumar, Rani Mohanraj, Stefan Listl, Samiksha Chopra, Vikrant Jadhav, Anika Arora, Chintan Valiya, Madhuri Pattamata

**Affiliations:** 1 Cleft and Craniofacial Department, Sree Balaji Dental College and Hospital, Bharath Institute of Higher Education and Research, Velachery Main Road, Chennai, Tamil Nadu 600100 India; 2 Bhagavan Mahaveer Jain Hospital, Bangalore, India; 3 Department of Orthodontics, University Medical Centre Groningen, University of Groningen, Groningen, The Netherlands; 4 Department of Orthodontics and Dentofacial Orthopedics, School of Dental Medicine/Medical Faculty, University of Bern, Bern, Switzerland; 5 Chesterfield Royal Hospital, Chesterfield Royal Hospital NHS Foundation Trust, Calow, England; 6 Jawaharlal Nehru Medical College, KLE Academy of Higher Education and Research, Belagavi, Karnataka India; 7 Sree Balaji Medical College and Hospital, Bharath Institute of Higher Education and Research, Chrompet, Chennai, India; 8 Shri Sharad Pawar Dental College and Hospital (SPDC), Wardha, Maharashtra India; 9 Yenepoya University: Yenepoya (Deemed to Be University), Mangaluru, Karnataka India; 10 Isha Hospital, Vadodara, Gurajat India; 11 John’s Research Institute, Bangalore, India; 12 Manav Rachna Dental College, Faridabad, Haryana India; 13 Amandeep Hospital, Amritsar, Punjab India; 14 Sant Parmanand Hospital, Delhi, India; 15 Vishwanath Katti Institute of Dental Sciences, KLE Academy of Higher Education & Research, Belagavi, India; 16 Samarth, Chennai, India; 17 Heidelberg University BIOMS: Universitat Heidelberg Bioquant, Heidelberg, Germany; 18 Radboud Universiteit Nijmegen: Radboud Universiteit, Nijmegen, The Netherlands

**Keywords:** Randomized controlled trials, Cleft lip and palate, Nasoalveolar moulding, NAM treatment, CLP, PSIO, Presurgical infant orthopaedics

## Abstract

**Background:**

Cleft lip and palate (CLP) are among the most common congenital anomaly that affects up to 33,000 newborns in India every year. Nasoalveolar moulding (NAM) is a non-surgical treatment performed between 0 and 6 months of age to reduce the cleft and improve nasal aesthetics prior to lip surgery. The NAM treatment has been a controversial treatment option with 51% of the cleft teams in Europe, 37% of teams in the USA and 25 of cleft teams in India adopting this methodology. This treatment adds to the already existing high burden of care for these patients. Furthermore, the supporting evidence for this technique is limited with no high-quality long-term clinical trials available on the effectiveness of this treatment.

**Method:**

The NAMUC study is an investigator-initiated, multi-centre, single-blinded randomized controlled trial with a parallel group design. The study will compare the effectiveness of NAM treatment provided prior to lip surgery against the no-treatment control group in 274 patients with non-syndromic unilateral complete cleft lip and palate. The primary endpoint of the trial is the nasolabial aesthetics measured using the Asher McDade index at 5 years of age. The secondary outcomes include dentofacial development, speech, hearing, cost-effectiveness, quality of life, patient perception, feeding and intangible benefits. Randomization will be carried out via central online system and stratified based on cleft width, birth weight and clinical trial site.

**Discussion:**

We expect the results from this study on the effectiveness of treatment with NAM appliance in the long term along with the cost-effectiveness evaluation can eliminate the dilemma and differences in clinical care across the globe.

**Trial registration:**

ClinicalTrials.gov CTRI/2022/11/047426 (Clinical Trials Registry India). Registered on 18 November 2022. The first patient was recruited on 11 December 2022. CTR India does not pick up on Google search with just the trial number. The following steps have to be carried out to pick up.

How to search: (https://ctri.nic.in/Clinicaltrials/advsearch.php—use the search boxes by entering the following details: Interventional trial > November 2022 > NAMUC).

**Supplementary Information:**

The online version contains supplementary material available at 10.1186/s13063-024-08229-z.

## Introduction

Cleft lip and palate (CLP) is among the most common congenital malformations with an overall incidence of around 1 in 700 newborns [1], and as a result, between 27,000 and 33,000 children in India are born with clefts every year [2]. CLP is accompanied by a wide variety of dental and skeletal anomalies, which have a long-term impact on the patient’s facial aesthetics, function and self-esteem.

One of the first treatments provided for these children is nasoalveolar moulding (NAM). This is a nonsurgical jaw-orthopaedic treatment for newborns with unilateral or bilateral cleft lip and alveolus with or without cleft palate, usually performed between 1 and 5 months of age to improve the position of the premaxilla and to reduce the size of the cleft width prior to lip surgery [3]. The treatment attempts to reposition the nasolabial and maxillary segments closer to each other and to mould the cartilages of the nose. The main objectives of the NAM therapy have been cited as (i) to reposition maxillary segments in a favourable anatomical position; (ii) to facilitate primary lip, alveolar and nasal surgeries; (iii) to reduce nasal deformity; (iv) to improve the projection of nasal tip; (v) to facilitate feeding; (vi) to increase the columella length; and (vii) to correct septal position [4–7].

The use of NAM as a treatment option has sparked controversy, with some centres embracing this approach while others are opposed. For instance, findings from the Eurocleft project, which surveyed 196 cleft teams, revealed that approximately half (51.7%) of the cleft teams in Europe implement some form of maxillary infant orthopaedics [8]. However, the UK has transitioned to a centralized care system for patients with orofacial clefts [9–11], with none of the centres currently employing NAM treatment. A survey conducted in 2011 across 117 centres in the USA found that 37% of cleft teams offered NAM treatment [12]. However, a subsequent survey showed that 68% of centres now provide NAM treatment [13]. In India, a recent survey indicated that 25% of centres are routinely conducting NAM treatment [14].

Although the above data shows that around 25 to 68% of cleft teams practise NAM routinely, the evidence to support this procedure is inconsistent. Several short-term studies on unilateral cleft lip, alveolus and palate (UCLAP) patients indicate that NAM treatment significantly improves nasal symmetry, reduces the severity of the cleft, reduces the need for other surgeries like lip/nose revision [4–7, 15–17], minimizes scarring and facilitates feeding [3, 16]. Other studies, however, show that NAM compromised future facial growth in addition to increased burden of care [18, 19]. The vast majority of studies, including a systematic review, report no difference in speech, facial growth and facial aesthetics of these children in the long term [2, 20–24]. The studies reported are of low quality, mainly retrospective in nature, mainly being single-centre and low sample size and lack an appropriate control group. There are no prospective long-term RCTs evaluating the effectiveness of the NAM therapy.

This randomized controlled trial aims to evaluate the long-term effectiveness of nasoalveolar moulding compared to no treatment on nasolabial aesthetics at 5 years in children with a non-syndromic complete unilateral cleft lip, alveolus and palate.

## Objectives

The NAMUC study will randomly allocate babies with UCLAP to either nasoalveolar moulding treatment followed by lip and palate surgery or directly to lip and palate surgery with no NAM treatment. Both groups will be followed up to 5 years of age.

The primary objective is to evaluate the effect of the NAM on nasolabial aesthetics by comparing with no NAM treatment. The secondary objectives are to investigate the effect of NAM on (i) dentofacial aesthetics, (ii) speech, (iii) hearing, (iv) quality of life, (v) cost effectiveness and (vi) intangible benefits.

## Trial design

**Fig. 1:**
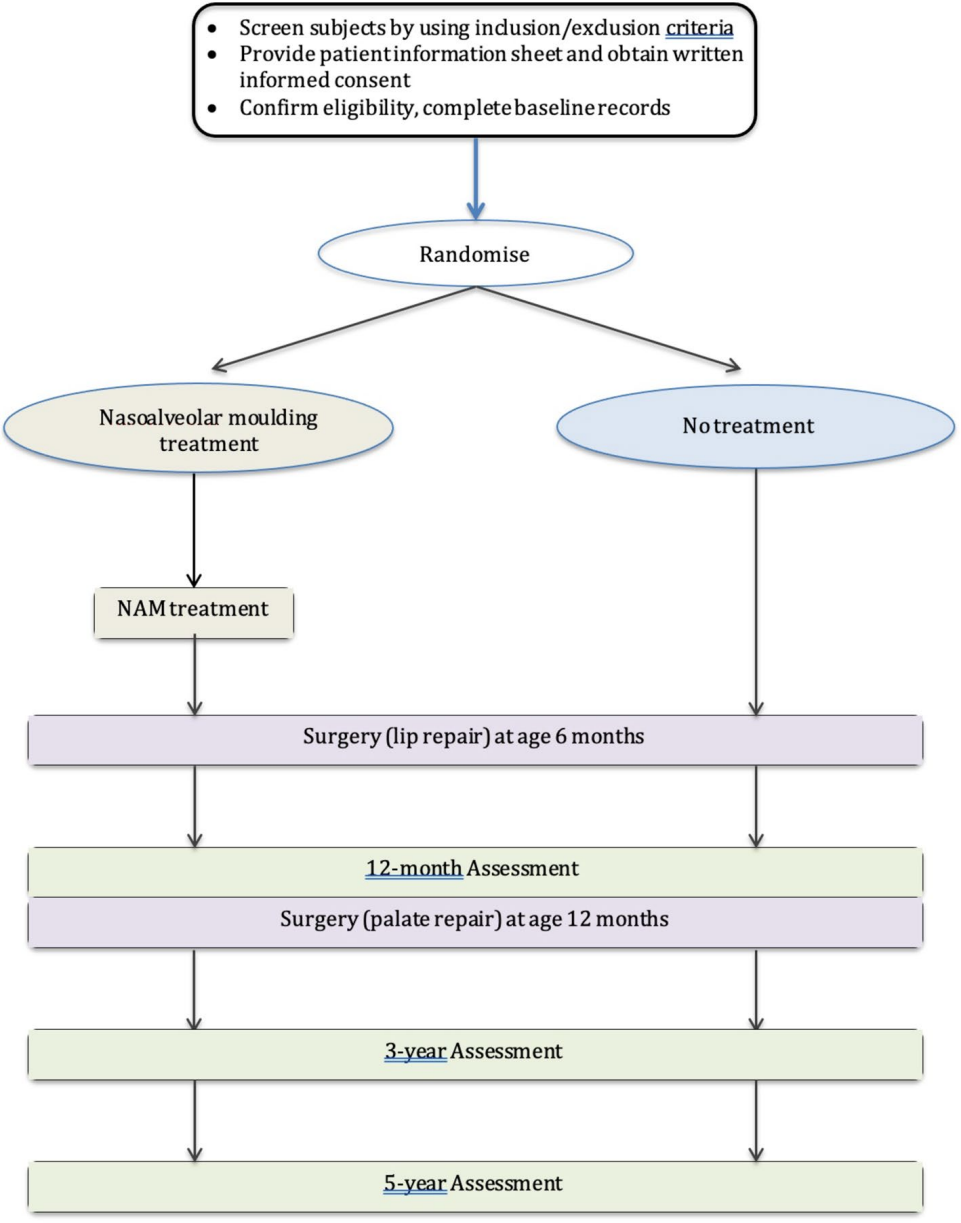
Schematics of trial design

The NAMUC study is a single-blinded, multi-centre randomized controlled superiority trial assessing the effects of NAM therapy, with a parallel group design and 1:1 allocation ratio. An overview of the trial design is shown in Fig. 1.

## Methods: participants, intervention and outcomes

### Study setting

This randomized controlled trial will be carried out at nine cleft lip and palate centres across India. Criteria for selection of participating centres are based on the volume of cases, experience with the NAM procedure and ability to enrol patients into a clinical trial as demonstrated with past research experience. The centres’ names and geographic locations are shown in Supplementary Table S1.

### Eligibility criteria

Newborn babies with UCLAP will be screened under the below inclusion and exclusion criteria.

Inclusion criteria:

- Infants with UCLAP
- No syndromes or other congenital anomalies
- Infants < 7 weeks old (corrected for gestational age)
- Medically fit for treatment
- One parent/guardian a native language speaker
- Signed informed consent

Exclusion criteria:

- Consent not obtained
- Bilateral, incomplete or submucous cleft or Simonart’s bands
- Congenital hearing loss or structural middle ear anomalies

### Intervention

Using the above-mentioned inclusion and exclusion criteria patients will be randomly allocated to nasoalveolar moulding treatment or to a no-treatment control group. The NAM group will have the appliance fitted with the aim of reducing the cleft width and improving nasolabial appearance. The no-treatment control group will not have any treatment until 6 months of age. At 6 and 12 months of age, patients from both groups will undergo surgery to correct the cleft lip and cleft palate, respectively. The patients will be followed up with routine care until 5 years of age.

### Selection of method for NAM

The method of NAM will be standardized for this trial. The Grayson’s technique, which is the technique followed by the majority of the collaborating centres will be followed [3]. Dr Grayson (Grossman School of Medicine, New York University, USA) will act as the lead clinician for the calibration of investigators involved in patient care. Dr Pedro Santiago (School of Medicine, Duke University, USA) will calibrate the orthodontists involved in the NAMUC study.

### Interventions: description

#### Nasoalveolar moulding treatment

The NAM treatment will involve taking a silicone impression of the maxillary arch and fabricating the appliance in acrylic (handmade). The appliance will have a uniform thickness of 1 mm with 3–4-mm thickness in the cleft alveolar region. The plate will have a stent to allow the attachment of elastics. The appliance will be secured with a denture adhesive paste and with adhesive strips, for, eg. 3 M Steri Strip (3M, Two Harbors, MN, USA). The elastics will be stretched on the lesser segment side to move the greater segment towards the cleft. Adjustments to move the greater segment will be carried out by trimming the acrylic in the cleft alveolar region around 2 to 3 mm every week. The aim of this procedure will be to align the greater and lesser segments of the maxillary arch into an ideal ‘U’ shaped arch form, thus minimizing the cleft width.

**Fig. 2:**
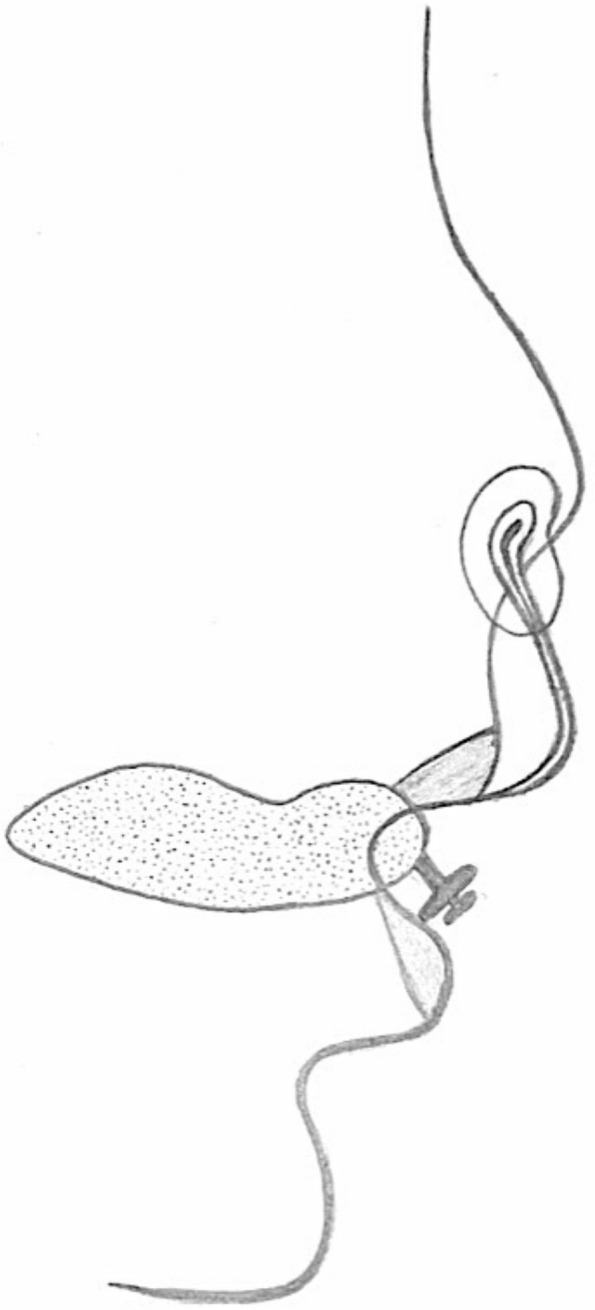
Nasoalveolar moulding appliance in situ with a nasal stent to lift the cleft-sided nostril upward and forward

When the alveolar cleft width is around 4 mm, a nasal stent will be added to lift the cleft-sided nostril upward and forward as illustrated in the picture (Fig. 2). The appliance will be in situ until the patient is ready for surgery.

### Selection of surgical method and timing

A standard surgical technique will be followed for all the patients taking part in the trial. The modified Millard technique for lip repair along with a rhinoplasty, and the Bardach two-flap technique for palatoplasty were agreed as the method of choice. The timing will be standardized to 6 months for lip repair and 12 months for palate repair. As these surgical methods and timings were already being followed at the majority of the included centres, it was decided to implement them in this trial as well. Dr Court Cutting (Langone Medical Centre, New York University, New York, USA) will be the lead clinician for the surgical calibration exercise.

### Concomitant care

Implementing nasoalveolar moulding treatment in non-syndromic patients with complete unilateral cleft lip, alveolus and palate or lip and palate surgery with no NAM treatment will not require alteration to usual care pathways (including use of any medication) and these will continue for both trial arms.

### Adherence

Clinicians and nurses will be available to guide patients during the intervention with NAM. Caregivers of patients in the control group will be contacted monthly to answer any questions that they may have.

A record will be made by the supervising clinician of participants’ adherence to NAM treatment. Patients who fail to continue with NAM treatment will be followed for further care, and to collect data during our predetermined follow-up time points, provided consent is obtained. Patients who fail to complete the trial at a later stage will be contacted and reasons for dropout will be recorded.

### Enrolment and randomization

Upon identification of a patient likely to satisfy the inclusion criteria, the Site Coordinator (SC) will be notified. The SC will primarily be the surgeon or the orthodontist. The SC will arrange for a screening and medical status assessment. If accepted, the SC will complete the screening log and enrol the patient in the NAMUC Trial. They will complete a log with the patient’s name, date of birth and screening number. This document will be held at the site and will not be provided to the Data Coordination Centre (DCC) to ensure patient-identifiable data is not transferred out of the clinical site.

The surgeon and orthodontist will assess the patient for inclusion and exclusion criteria. If the patient is identified as a potential trial participant, the parent/guardian will be invited to join, and information will be provided about the trial and the process of enrolment. The patient information sheet and consent form will be provided to the parent/legal guardian, and they will then be given ample time to read and understand the forms and to ask any questions.

If the parent/guardian agrees to take part, copies of the signed forms will be made and one given to the parents, one sent to DCC and the original retained in the patient notes.

There will be no cost to participate in this research study. If required, parking and travel costs will be reimbursed. Further strategies to promote recruitment will include conducting camps in neighbouring areas and a study leaflet for referrers, other cleft patients and participants.

#### Randomization

Randomization will be performed with an interactive web response system. The sequence generation was developed by an independent member at the Kings College Clinical Trials Unit (Kings College London, UK). Patients will be randomized in a 1:1 ratio and stratified on cleft width (< 8 mm, between 8 and 12 mm or ≥ 12 mm), birth weight (< 2750 g or ≥ 2750 g) and clinical trial site.

Two members from each trial site will be given an online user ID and password for the online randomization system.

Once the consent is obtained and the forms completed, the participant data will be entered into the central online randomization system and the participant will be randomized using the secure online randomization system. Each participant will be assigned a unique randomization number.

#### Participant withdrawal

Should a parent/guardian choose to withdraw the baby from the trial at any point, the child might receive a different NAM or surgical procedure than the one initially allocated in the trial. They will be required to sign the withdrawal form and no further data will be collected for trial purpose.

The NAMUC trial will exclude all syndromic patients. However, some syndromes may not be identified soon after birth. If patients are identified as having a syndrome after randomization (if signs and symptoms show up later), they will remain in the study and continue with follow-up appointments.

#### Sample size estimation

We have based the sample size calculation from a pilot on patients treated with NAM and without NAM. The data was collected at 5 years of age and evaluated for the primary outcome, facial aesthetics evaluated using the Asher-McDade index score [25]. For a mean difference of 0.06, an effect size of 0.44, a power of 90% and an alpha of 0.05, we need a sample of 109 per group (total 218 patients), and for an estimated non-compliance rate of 20%, the total sample size required is 274.

### Trial interventions

#### Introduction

Participants randomized to the ‘NAM’ group of the NAMUC study will receive NAM treatment presurgically, 6-month lip surgery and 12-month palate surgery.

Participants randomized to the ‘No NAM’ group will not receive any NAM treatment but will have 6-month lip surgery and 12-month palate surgery. The trial overview is shown in Fig. 1.

#### NAM treatment

Each NAM treating clinician will be provided with written descriptions of the clinical procedure and a video prepared by the expert illustrating all the steps that must be followed. They will receive this information 4 weeks prior to the calibration exercise and will attend the calibration exercise conducted by the expert.

#### Surgical technique

The surgeons taking part in the NAMUC trial will be provided with written instruction and a video of both lip and palate surgical protocols. This information will be provided 4 weeks prior to the calibration exercise and all surgeons will attend the calibration exercise conducted by the expert.

#### Blinding

It is not possible to blind clinicians involved in the NAMUC study, as they conduct interventional clinical procedures (open-label). However, every effort will be made to collect durable records, so that raters can be blinded for assessment of outcomes. These include the primary and all the secondary outcomes, assessed at age 5. For quality of life assessment, the interviewers and assessors will be independent persons not involved in the study; however, a full blinding would not be possible due to the nature of the data. The data analysts will be blinded for the treatment groups.

#### Calibration

Prior to starting the trial, a formal process of both NAM and surgical standardization will take place for each research team. There will be written and video instructions from the expert along with seminars and discussions when needed. A delegation log will be signed by all the trial surgeons and NAM clinicians and countersigned by lead clinicians.

An intermediate NAM and surgical calibration exercise at 18 months and 3 years from the start of the trial will be arranged to make sure the orthodontists and surgeons stick to standardized techniques and no deviations of the protocol occur.

Calibration for speech assessments for speech and language therapists from each centre will be held prior to the data collection stage. A series of audio recordings of infants, not involved in the trial, will be used for calibration. The age groups of infants for practice recordings will match the data collection points in the study. A core group will be set up prior to the calibration exercise. The speech recordings will be assessed for quality by the core group and appropriate training will be provided where needed.

### Outcomes

The primary and secondary outcomes will be collected at specified time points that include:

- *T1*: Baseline
- *T2*: Post NAM therapy/pre-lip surgical closure
- *T3*: Post lip repair—3 weeks post-surgery
- *T4*: 12 months
- *T5*: Post Palate surgical closure—3 weeks post-surgery
- *T6*: 3 years
- *T7*: 5 years

At all time points study models, intra- and extra-oral photographs will be taken.

3D stereophotogrammetry records will be taken at all time points in the centres that have access to this facility.

Post-operative complications will be assessed at 24 h, 48 h and 7 and 30 days. Secondary/revision surgery due to failure/dehiscence will also be assessed.

**Fig. 3:**
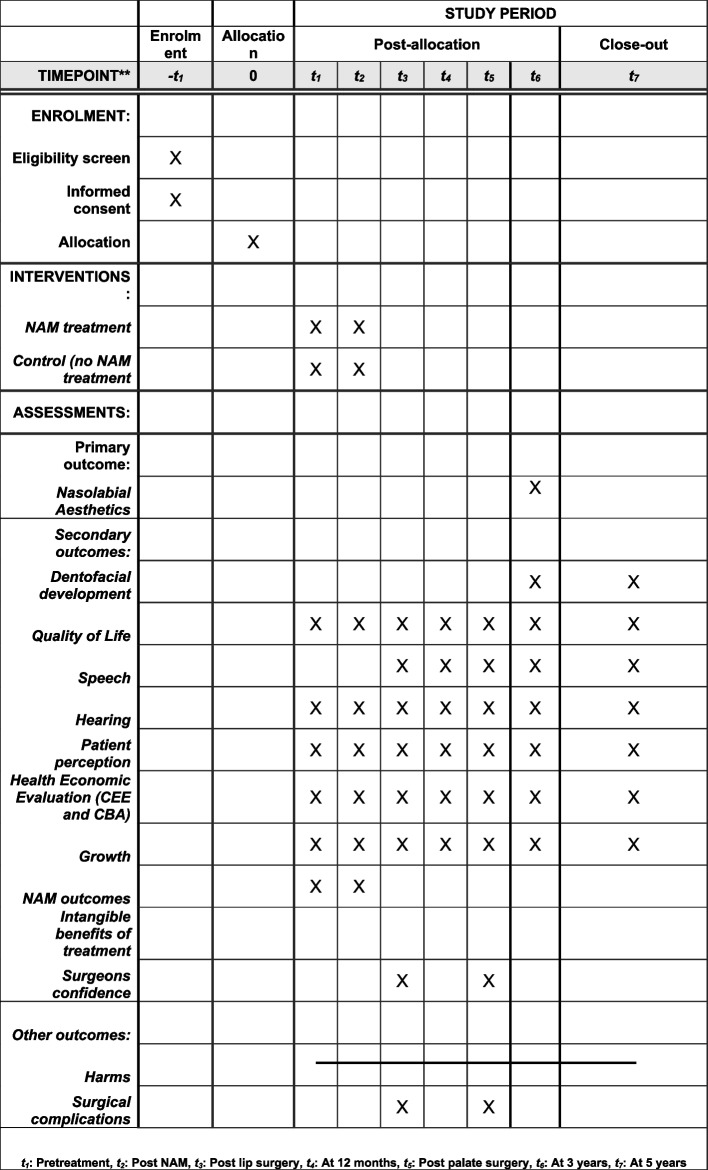
SPIRIT figure

A summary of all the outcomes and timeline is reported in Table 1 and Fig. 3.

**Table 1:** Outcomes at each time point

**Outcomes**	**At 5 years (T7)**	**At 3 years (T6)**	**Post-palate repair (T5)**	**At 12 months (T4)**	**Post-lip repair (T3)**	**Post-NAM therapy (T2):**	**Baseline (T1)**
***Dentofacial development***	a. 5-year-old index on dental casts b. Soft tissue ANB from profile photograph c. Skeletal ANB from lateral cephalogram d. Facial aesthetics measured according to Asher-McDade index e. 3D facial aesthetic assessment using stereophotogrammetric images	a. Facial aesthetics measured according to Asher-McDade index b. 3D facial aesthetic assessment using stereophotogrammetric images					
***Speech***	a. Velopharyngeal insufficiency score (score scale from 0 to 6 and ≥ 4 will be considered insufficient) b. Velopharyngeal composite summary score c. Articulation d. The Intelligibility in Context Scale questionnaire (ICS)	a. Velopharyngeal insufficiency score (score scale from 0 to 6 and ≥ 4 will be considered insufficient) b. Velopharyngeal composite summary score c. Articulation d. The Intelligibility in Context Scale questionnaire (ICS)		a. Canonical babbling			
***Hearing***	a.Pure tone audiometry b. Flat line tympanogram	a. Pure tone audiometry b. Flat line tympanogram	a. Pure tone audiometry b. Flat line tympanogram	a. Pure tone audiometry b. Flat line tympanogram	a. Pure tone audiometry b. Flat line tympanogram	a. Pure tone audiometry b. Flat line tympanogram	a. Pure tone audiometry b. Flat line tympanogram
***Quality of Life***	a. Parent Questionnaire: Parent’s perception of treatment questionnaire (PHQ-9) and Generalized Anxiety and Depressive Symptoms Scale	a. Parent Questionnaire: Parent’s perception of treatment questionnaire (PHQ-9) and Generalized Anxiety and Depressive Symptoms Scale	a. Parent Questionnaire: Parent’s perception of treatment questionnaire (PHQ-9) and Generalized Anxiety and Depressive Symptoms Scale	a. Parent Questionnaire: Parent’s perception of treatment questionnaire (PHQ-9) and Generalized Anxiety and Depressive Symptoms Scale	a. Parent Questionnaire: Parent’s perception of treatment questionnaire (PHQ-9) and Generalized Anxiety and Depressive Symptoms Scale	a. Parent Questionnaire: Parent’s perception of treatment questionnaire (PHQ-9) and Generalized Anxiety and Depressive Symptoms Scale	a. Parent Questionnaire: Parent’s perception of treatment questionnaire (PHQ-9) and Generalized Anxiety and Depressive Symptoms Scale
***Patient perception***	Care giver interviews	Care giver interviews	Care giver interviews	Care giver interviews	Care giver interviews	Care giver interviews	Care giver interviews
***Health Economic Evaluation (CEE and CBA)***	Cost evaluation form	Cost evaluation form	Cost evaluation form	Cost evaluation form	Cost evaluation form	Cost evaluation form	Cost evaluation form
***Intangible benefits of treatment***	Interviews	Interviews					Interviews
***Growth***	a. Weight b. Height	a. Weight b. Height	a. Weight b. Height	a. Weight b. Height	a. Weight b. Height	a. Weight b. Crown heel length c. Occipitofrontal circumference	a. Weight b. Crown heel length Occipitofrontal circumference
***NAM outcomes***						a. Interlabial width b. Interlalveolar cleft width c. Number of visits d. Duration of treatment e. Complications/harms/side effects f. Feeding diary	a. Interlabial width b. Interalveolar cleft width c. Feeding diary
***Surgeons’ confidence***			a. Questionnaire		a. Questionnaire		

Impressions for study models and photographs will be taken at all time points

#### Primary outcome

The primary endpoint for the study is at 5 years of age (at T7). The nasolabial aesthetic score using the Asher McDade index will be the primary outcome of this study. The Asher McDade index is a 5-point validated index used to evaluate the nasolabial aesthetics in patients with UCLAP. This 5-point ordinal scale measures the aesthetics of nasal form, vermilion border, nasal symmetry and nasal profile using 2D photographs.

#### Secondary outcomes

Dentofacial outcomes:

- Five-year-old index from study models
- Soft tissue ANB from profile photograph
- Skeletal change (ANB) from lateral cephalometry

Speech:

- Velopharyngeal insufficiency score (score scale from 0 to 6 and ≥ 4 will be considered insufficient)
- Velopharyngeal composite summary score
- Articulation
- The Intelligibility in Context Scale questionnaire (ICS)

Hearing:

- Pure tone audiometry
- Flat line tympanogram

Quality of life:

- Qualitative interviews
- Parent Questionnaire: Parent’s perception of treatment questionnaire (PHQ-9) and Generalized Anxiety and Depressive Symptoms Scale

Health economics:

- Cost analysis/ Full economic evaluation

Feeding:

- Quantity of milk intake during the first 6 months
- Height and weight gain

Intangible benefits:

Impact of the trial on researcher development, professional development of clinical staff and organizational capacity and delivery of clinical services

### Participant timeline

**Fig. 4:**
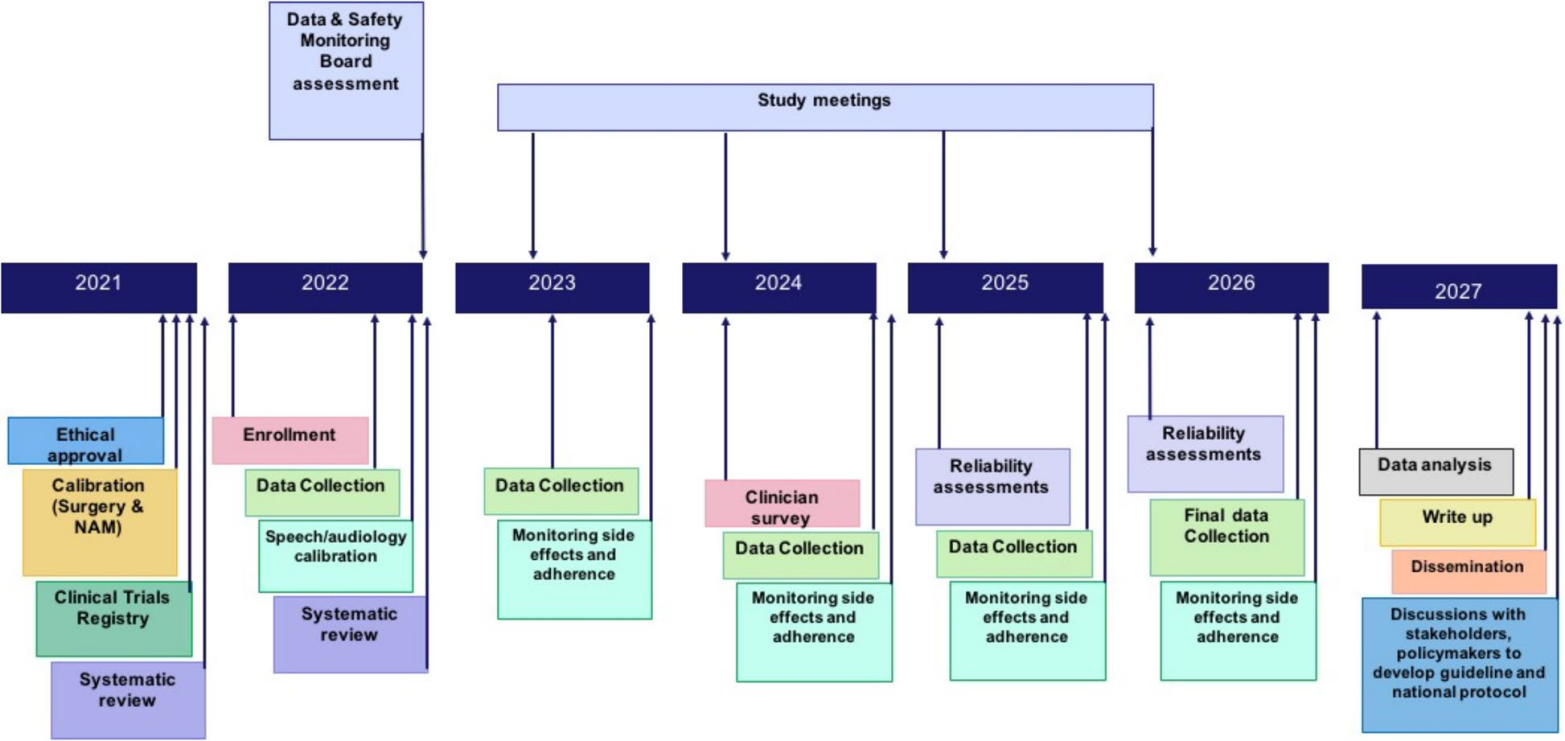
The schedule of eligibility screening, enrollment, group allocation, visits, interventions and assessments

The schedule of eligibility screening, enrollment, group allocation, visits, interventions and assessments are shown in Fig. 4.

### Plans for collection, laboratory evaluation and storage of biological specimens

Not applicable, the trial does not involve collecting biological for storage.

### Data analysis

A summary of data analysis is included. A full plan will be developed prior to the final analysis of the trial data. This will be agreed upon by the trial steering committee and independent data monitoring board prior to analysis.

#### Statistics: outcomes

A detailed statistical plan will be developed prior to the final analysis of the trial. An overall outline of the statistical plan is reported here.

The principle of ‘intention to treat’ analysis will be performed for primary analysis for comparing end outcomes between two groups. Also, analysis will be performed on the ‘as treated’ principle. Data imputation will be done for the primary outcome if the missing values are greater than 10%. ANCOVA or multiple-regression analysis will be used to compare the outcomes between groups at the end of 5 years adjusting for baseline outcome variables. In addition, a mixed model analysis will be used to find out changes that occur over time.

Categorical variables will be carried out using Pearson’s chi-square statistics or logistic regression. Categorical data will be given as mean differences or odds ratios with 95% confidence intervals (95% CI). Multiple-linear or logistic regression will be used to predict factors associated.

The statistical significance will be kept at 0.05 for all analysis.

#### Research ethics approval

The trial will follow the principles of the Declaration of Helsinki and the Indian Council of Medical Research (ICMR). Ethical approval was obtained from the Institutional Ethics Committee, BIHER (SBDCH/IE/06/2021/03), and from each participating centre’s Ethical Board.

#### Confidentiality

All case report forms (CRFs) will have a unique patient ID allocated upon randomization. The DCC will preserve the confidentiality of the participants taking part in the trial. The Indian Data Protection Law 2019 and all Indian Council of Medical Research (ICMR) guidelines will be followed in this trial.

#### Ancillary and post-trial care

There is no anticipated harm and compensation for trial participation. Owing to the nature of the trial, there are no provisions for post-trial care.

## Monitoring

### Trial Management Group

The Trial Management Group (TMG) comprises members of the DCC, administrative members and members of the core research group. This group is responsible for day to day running of the trial.

### Trial Steering Committee

A Trial Steering Committee (TSC) will be established according to ICMR guidelines and will be chaired by a senior academic clinician. The TSC will include trial investigators, members of the trial team at DCC, administrative members, a CLP patient and a lay person in addition to an independent expert. The independent cleft expert for the trial will be Professor Anne Marie Kuijpers Jagtman (University Medical Center Groningen, Groningen, The Netherlands). The role of the Trial Steering Committee is to provide overall supervision for the trial and provide advice through its independent Chairperson.

### Data monitoring: formal committee

#### Data and Safety Monitoring Board

The Data and Safety Monitoring Board (DSMB) will comprise a chair, a statistician, an expert orthodontist and an expert surgeon. The DSMB will be responsible for reviewing and assessing recruitment, interim monitoring of safety, trial conduct and external data.

The DSMB board comprises the following members: Dr Thara Rangaswamy (Psychiatrist and Vice President, Schizophrenia Research Foundation, India) (chair), Dr Deborah Sybil (Maxillofacial Surgeon, Jamia Millia Islamia University, New Delhi) (surgeon), Dr Vadivel Kumar (Orthodontist, Vinayaka Mission Research Foundation) (orthodontist), Dr Saravana Kumar (Scientist C, ICMR National Institute of Epidemiology (NIE)) (statistician). When 50% of recruitment is complete, the independent DSMB will carry out a preplanned interim safety analysis. The DSMB will receive information on recruitment, data quality, protocol compliance, missing data, surgical and orthodontic complications, intensive care unit and hospital mortality for recruited participants. Following the interim analysis, the DSMB will submit the report to the sponsor with a recommendation of one of the following: (i) continue as planned, (ii) early discontinuation due to harm or (iii) a protocol change. The sponsor will have the final decision-making responsibility.

### Trial Site Monitoring

The principal investigator (PI) at each site will be responsible for assuring compliance with the protocol. On-site visits will be conducted throughout the trial to ensure adherence to the protocol and to protect the rights and safety of the participants. A monitoring report will be prepared and presented to the PI at the site and to the Trial Management Group.

As a funder, the Department of Biotechnology (DBT), India, may also audit the trial as necessary.

### Auditing

The funder or the sponsor, with an independent auditor, can audit the trial at any time and at any study site. All parts of the trial including protocol compliance, consent process, data management, etc., can be audited.

### Protocol amendments

Any substantial protocol amendments will be re-evaluated by the ethical committees and amendments reported to all relevant parties including the sponsor, all committees, investigators and trial participants and trial registry.

### Harms

#### Adverse events

An adverse event (AE) is defined as any untoward incident in a patient to whom an intervention has been administered, including events that are not necessarily caused by or related to the procedure.

#### Serious adverse events

A serious adverse event (SAE) is defined as any untoward medical occurrence that meets any of the five criteria as set out in the National Institute of Dental and Craniofacial Research (NIDCR) website.

#### Unanticipated problems

The unanticipated problems (UP) involving risks to subjects or others include, in general, any incident, experience or outcome that meets all of the three criteria as set out in the NIDCR website.

Any incident that meets any of the three criteria will require consideration for substantive changes to the protocol. This is to protect the safety of the participants or others involved in the trial. Any such incident will be recorded and reported throughout the study.

#### Severity or grading of adverse incidents

The grading/ severity should be made by the investigator responsible for the clinical care of the patient. The five criteria from ‘mild’ to ‘death’ as set out in NIDCR will be used for grading.

#### Follow-up of adverse events

All adverse events should be followed until satisfactory resolution or until the investigator responsible for the care of the participant deems the event to be chronic or the patient is stable. The six criteria (recommended by NIDCR) will be applied by the investigator.

#### Reporting procedures

**Fig. 5:**
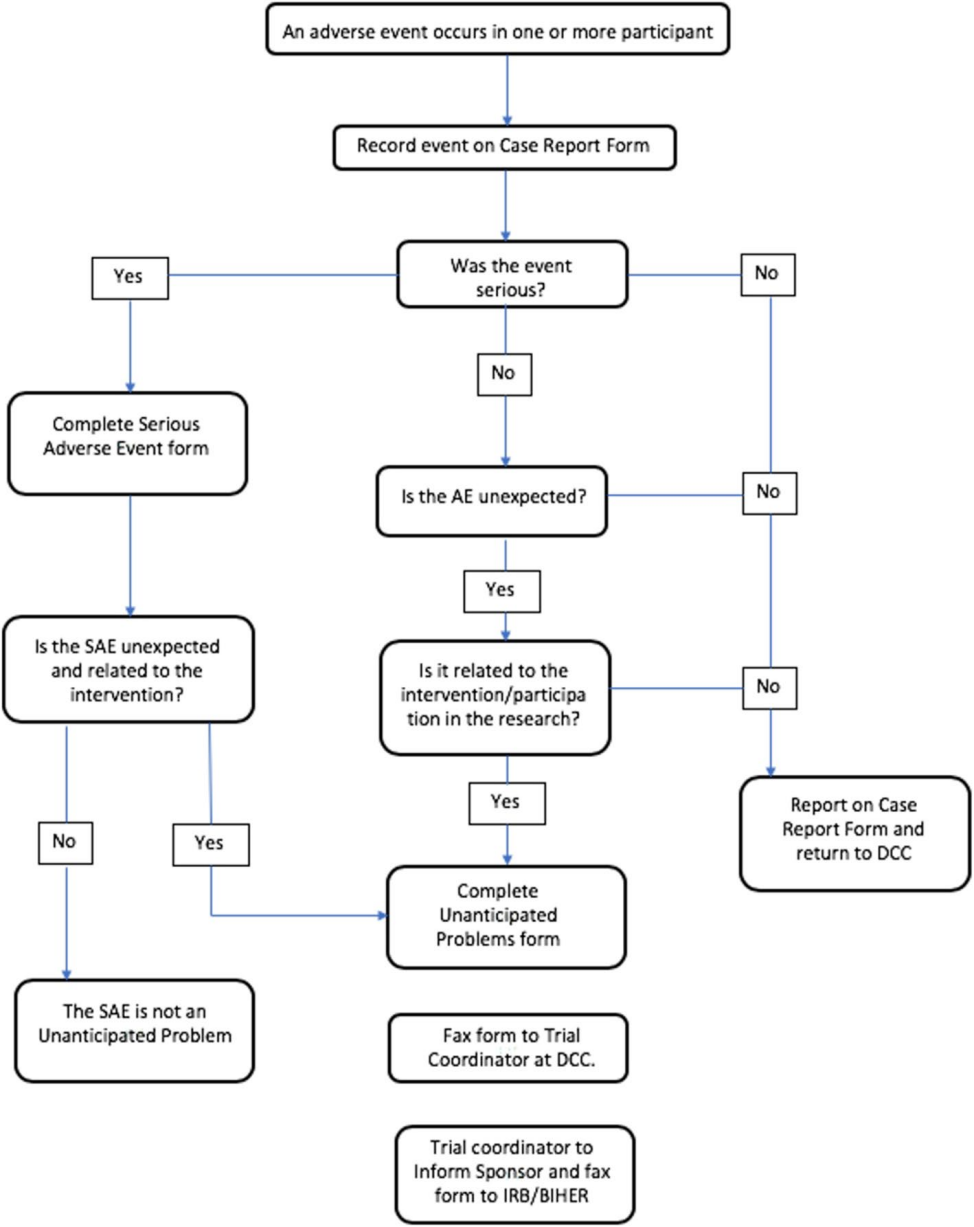
Flowchart for adverse event reporting

The principal investigator will report all AE, SAE and UP that are observed during NAM treatment and during and 30 days post-surgery. A flowchart is given to help in determining the reporting requirements (Fig. 5).

The DCC will be responsible for reporting SAE and UP. A final report will be provided with all details to the sponsor and a copy to the Institutional Review Board.

The reporting deadlines will be 1 week for unanticipated problems and 15 days for serious adverse events.

### Dissemination policy

The study will be disseminated to the three important stakeholders: (i) scientific community, (ii) patients with cleft and (iii) general public.

i. Scientific community
  The results of this project will be published in peer-reviewed journals with open access policies. The timeline for this is up to 12 months after the last participant completes the study (T7). In addition, the results will be presented at international and national scientific conferences.
ii. Individuals with cleft
  All educational materials written in lay format will be made available for free on our trial website. In addition, these will be provided to the NGOs and government organizations funding cleft care in India for advertising on their websites. Participants involved in the trial will be invited to attend a workshop and will be informed of the results. They will also be asked to disseminate the results to other patient groups.
iii. General public
  The Chief Investigator will reach out to journalists and other media with a plan to write a feature article on the study results.

## Trial status

The study protocol (Version 4.14, 20 March 2022) was approved by the BIHER institutional ethics committee in March 2022. The first patient was recruited on 11 December 2022. As of 21 December 2023, 90 babies had been recruited. The recruitment is now ongoing and is expected to be complete by March 2025.

### Supplementary Information

Supplementary Material 1.

## Data Availability

All study authors/sites will have access to the database after the inclusion of the last patient. The details regarding confidentiality, data protection, intellectual property and publication are documented in a clinical trial agreement.

## Abbreviations

AE: Adverse event
CI: Chief Investigator
CF: Consent form
CRF: Case report form
DCC: Data Coordinating Centre
DSMB: Data and Safety Monitoring Board
GCP: Good Clinical Practice
ICS: Intelligibility in Context Scale questionnaire for parents
IEC: Institutional Ethical Committee
NAM: Nasoalveolar moulding
NAMUC: Nasoalveolar moulding in complete unilateral non-syndromic cleft patients
NIDCR: National Institute of Dental and Craniofacial Research
PI: Principal Investigator
RCT: Randomized controlled trial
SAE: Serious adverse event
SC: Site Coordinator
TC: Trial Coordinator
TMF: Trial Master File
TMG: Trial Management Group
TSC: Trial Steering Committee
UCLP: Unilateral Cleft Lip and Palate
UCLAP: Unilateral cleft lip, alveolus and palate
UP: Unanticipated problem

## References

[1] Vanderas AP (1987). Incidence of cleft lip, cleft palate, and cleft lip and palate among races: a review. Cleft Palate J.

[2] Mossey P, Little J (2009). Addressing the challenges of cleft lip and palate research in India. Indian J Plast Surg.

[3] Grayson BH, Santiago PE, Brecht LE, Cutting CB (1999). Presurgical nasoalveolar molding in infants with cleft lip and palate. Cleft Palate Craniofac J.

[4] Barillas I, Dec W, Warren SM, Cutting CB, Grayson BH (2009). Nasoalveolar molding improves long-term nasal symmetry in complete unilateral cleft lip-cleft palate patients. Plast Reconstr Surg.

[5] Ezzat CF, Chavarria C, Teichgraeber JF, Chen JW, Stratmann RG, Gateno J, Xia JJ (2007). Presurgical nasoalveolar molding therapy for the treatment of unilateral cleft lip and palate: a preliminary study. Cleft Palate Craniofac J.

[6] Lee CT, Grayson BH, Cutting CB, Brecht LE, Lin WY (2004). Prepubertal midface growth in unilateral cleft lip and palate following alveolar molding and gingivoperiosteoplasty. Cleft Palate Craniofac J.

[7] Grayson BH, Cutting C, Wood R (1993). Preoperative columella lengthening in bilateral cleft lip and palate. Plast Reconstr Surg.

[8] Shaw WC, Semb G, Nelson P, Brattstrom V, Molsted K, Prahl-Andersen B, Gundlach KK (2001). The Eurocleft project 1996–2000: overview. J Craniomaxillofac Surg.

[9] Bearn D, Mildinhall S, Murphy T, Murray JJ, Sell D, Shaw WC, Williams AC, Sandy JR (2001). Cleft lip and palate care in the United Kingdom–the Clinical Standards Advisory Group (CSAG) study. Part 4: outcome comparisons, training, and conclusions. Cleft Palate Craniofac J.

[10] Sandy J, Williams A, Mildinhall S, Murphy T, Bearn D, Shaw B, Sell D, Devlin B, Murray J (1998). The Clinical Standards Advisory Group (CSAG) cleft lip and palate study. Br J Orthod.

[11] Sandy JR, Williams AC, Bearn D, Mildinhall S, Murphy T, Sell D, Murray JJ, Shaw WC (2001). Cleft lip and palate care in the United Kingdom–the Clinical Standards Advisory Group (CSAG) study. Part 1: background and methodology. Cleft Palate Craniofac J.

[12] Sischo L, Chan JW, Stein M, Smith C, van Aalst J, Broder HL (2012). Nasoalveolar molding: prevalence of cleft centers offering NAM and who seeks it. Cleft Palate Craniofac J.

[13] Avinoam SP, Kowalski HR, Chaya BF, Shetye PR (2022). Current presurgical infant orthopedics practices among American cleft palate association-approved cleft teams in North America. J Craniofac Surg.

[14] Batra P, Sybil D, Izhar A, Batra P, Thiruvenkatachari B (2023). Standard of care for patients with cleft lip and palate in India—a questionnaire-based study. Cleft Palate Craniofac J.

[15] Jaeger M, Braga-Silva J, Gehlen D, Sato Y, Zuker R, Fisher D (2007). Correction of the alveolar gap and nostril deformity by presurgical passive orthodontia in the unilateral cleft lip. Ann Plast Surg.

[16] Maull DJ, Grayson BH, Cutting CB, Brecht LL, Bookstein FL, Khorrambadi D, Webb JA, Hurwitz DJ (1999). Long-term effects of nasoalveolar molding on three-dimensional nasal shape in unilateral clefts. Cleft Palate Craniofac J.

[17] Chou PY, Hallac RR, Ajiwe T, Xie XJ, Liao YF, Kane AA, Park YJ (2017). The role of Nasoalveolar molding: a 3D Prospective analysis. Sci Rep.

[18] Kornbluth M, Campbell RE, Daskalogiannakis J, Ross EJ, Glick PH, Russell KA, Doucet JC, Hathaway RR, Long RE, Sitzman TJ (2018). Active presurgical infant orthopedics for unilateral cleft lip and palate: intercenter outcome comparison of Latham, modified McNeil, and nasoalveolar molding. Cleft Palate Craniofac J.

[19] Levy-Bercowski D, Abreu A, DeLeon E, Looney S, Stockstill J, Weiler M, Santiago PE (2009). Complications and solutions in presurgical nasoalveolar molding therapy. Cleft Palate Craniofac J.

[20] Clark SL, Teichgraeber JF, Fleshman RG, Shaw JD, Chavarria C, Kau CH, Gateno J, Xia JJ (2011). Long-term treatment outcome of presurgical nasoalveolar molding in patients with unilateral cleft lip and palate. J Craniofac Surg.

[21] Prahl C, Kuijpers-Jagtman AM, Van’t Hof MA, Prahl-Andersen B (2003). A randomized prospective clinical trial of the effect of infant orthopedics in unilateral cleft lip and palate: prevention of collapse of the alveolar segments (Dutchcleft). Cleft Palate Craniofac J.

[22] Prahl C, Kuijpers-Jagtman AM, van’t Hof MA, Prahl-Andersen B (2001). A randomised prospective clinical trial into the effect of infant orthopaedics on maxillary arch dimensions in unilateral cleft lip and palate (Dutchcleft). Eur J Oral Sci.

[23] Rubin MS, Clouston SAP, Esenlik E, Shetye PR, Flores RL, Grayson BH (2019). Midface growth in patients with unilateral cleft lip and palate treated with a nasoalveolar molding protocol. J Craniofac Surg.

[24] Papadopoulos MA, Koumpridou EN, Vakalis ML, Papageorgiou SN (2012). Effectiveness of pre-surgical infant orthopedic treatment for cleft lip and palate patients: a systematic review and meta-analysis. Orthod Craniofac Res.

[25] Asher-McDade C, Roberts C, Shaw WC, Gallager C (1991). Development of a method for rating nasolabial appearance in patients with clefts of the lip and palate. Cleft Palate Craniofac J.

