# Supplementary material for: A multi-centric, single-blinded, randomized, parallel-group study to evaluate the effectiveness of nasoalveolar moulding treatment in non-syndromic patients with complete unilateral cleft lip, alveolus and palate (NAMUC study): a study protocol for a randomized controlled trial
Source: Trials. 2024 Jul 4;25:453. doi: 10.1186/s13063-024-08229-z (PMC11223389; doi:10.1186/s13063-024-08229-z)
Supplement: Supplementary file 1 — Supplementary Material 1. [file 13063_2024_8229_MOESM1_ESM.docx]

**Study sites**

| Centre ID | Centre | Location | Type of centre |
| --- | --- | --- | --- |
| 01 | Amandeep Hospital | Amritsar, Rajasthan | Secondary care hospital |
| 02 | Sharad Pawar Dental College | Wardha, Maharashtra | Academic institution/Tertiary care |
| 03 | Yenapoya Medical and Dental Colleges | Mangalore, Karnataka | Academic institution/tertiary care |
| 04 | Mahaveer Jain Hospital | Bengaluru, Karnataka | Secondary care hospital |
| 05 | KLE Academy of Higher Education and Research | Belgaum, Karnataka | Academic institution/tertiary care |
| 06 | Isha Hospital | Vadodara, Gujrat | Secondary care hospital |
| 07 | Sant Parmanand Hospital | Delhi | Secondary care hospital |
| 08 | Indira Gandhi Institute of Medical Sciences | Patna, Bihar | Academic institution/tertiary care |
| 09 | Sree Balaji Medical and Dental Colleges. | Chennai, Tamil Nadu | Academic institution/tertiary care |
